# A dwell position verification method for high dose rate brachytherapy

**DOI:** 10.1120/jacmp.v5i1.1944

**Published:** 2004-05-25

**Authors:** Lizhong Liu, Satish C. Prasad, Daniel A. Bassano, Joel Heavern, Bonnie Keshler, Seung S. Hahn

**Affiliations:** ^1^ Department of Radiation Oncology SUNY Upstate Medical University 750 East Adams Street Syracuse New York 13210

**Keywords:** HDR brachytherapy, dwell position verification, misadministration

## Abstract

Misplacement of dwell positions is a potential source of misadministration in high dose rate (HDR) brachytherapy. In this work, we present a dwell position verification method using fluoroscopic images. A mobile C‐arm fluoroscopic machine is used to take a snapshot of the treatment machine's check cable as it reaches the most distal dwell position. This fluoroscopic image is displayed side‐by‐side with a treatment planning image on a dual monitor relay station at the HDR treatment console. Any discrepancy between the check cable's position on the verification image and the intended dwell position on the planning image can be identified, immediately, thus avoiding the possibility of treating the wrong target volume.

PACS numbers: 87.53.Jw, 87.53.Xd, 87.59.Ci

## I. INTRODUCTION

In high dose rate (HDR) brachytherapy, the treatment dose to a target site is delivered by leaving a high activity Ir192 source at each dwell position for a specific amount of dwell time. The dwell positions and dwell times are determined during the treatment planning process, and they are programmed into the HDR treatment machine. Human and computer errors during the treatment planning and treatment preparation process can, potentially, place the dwell positions in the wrong location, resulting in treating the wrong target volume. Such incidents are rare, but they do occur, as evidenced by the US Nuclear Regulatory Commission (USNRC)'s event notification reports (see, for example, the USNRC website at http://www.nrc.gov ‐ searching for “HDR” in the “Event and Status Reports” collection gives the event reports) and other reports in the literature.^(1)^ It is, therefore, desirable to have an independent method available that allows for the verification of dwell positions before treatment delivery.

Several authors have investigated different methods of verifying and recording the dwell positions in HDR brachytherapy treatments. Sheikh‐Bagheri and Munro^2^ evaluated the possibility of monitoring the high activity Ir192 source using x ray fluoroscopic images. They suggested that use of a large air gap between the patient and the x ray image intensifier, a well designed anti‐scatter grid to suppress the spurious signals generated by the Ir192 γ rays, and a high current x ray fluoroscopy technique can make the real time monitoring of source position feasible. Duan et al.^(3)^ used a pinhole camera to capture the autoradiographic image of the active Ir192 source. However, images produced with these methods were either of poor quality^(2)^ or contained no patient anatomic information.^(3)^

In this work, we present a different method of verifying the dwell positions using x ray fluoroscopic images. A mobile C‐arm fluoroscopic machine is used to capture a fluoroscopic image of the check cable of a HDR treatment machine when it reaches the most distal dwell position during a treatment. The use of the check cable, instead of the active Ir192 source, produces a fluoroscopic image free of noise signals. This verification image is compared with the treatment planning image, both of them displayed on a dual monitor relay station, at the HDR treatment console. A difference between the position of the check cable and the intended first dwell position on the planning image can be easily identified, thus avoiding the treatment of a wrong site. Such a verification method requires minimum capital investment if a C‐arm is available, and it can be easily implemented in a clinic.

## II. MATERIALS AND METHODS

The mobile C‐arm fluoroscopic machine used in this study was described in an earlier publication.^(4)^ It is a model OEC 9800 surgical C‐arm (manufactured by GE Medical Systems Waukesha, Wisconsin). Its image intensifier (II) has a diameter of 12 inches, featuring a 1k×1k digital resolution. The C‐arm workstation is Ethernet ready, allowing the direct transfer of fluoroscopic images to a treatment planning computer, through a local area network. The dual monitors of the workstation allow two images to be displayed side‐by‐side, making image comparison a simple task. This mobile C‐arm machine has been in extensive clinical use at our institution, for taking orthogonal images for treatment planning of endobronchial and intracavitary HDR brachytherapy.^(4)^

The HDR treatment machine used in this study is a Varian VariSource 200 HDR unit (Varian Medical Systems, Inc., Palo Alto, California). It has a high activity Ir192 source attached to the end of a drive cable and an indexing system for 20 channels. A dummy cable is installed in the machine, and is used for checking the pathway from the machine safe to the intended dwell positions in the implant catheters. The check cable is a standard feature in all commercial HDR treatment machines, and a check run is performed before each treatment delivery.

**Figure 1:**
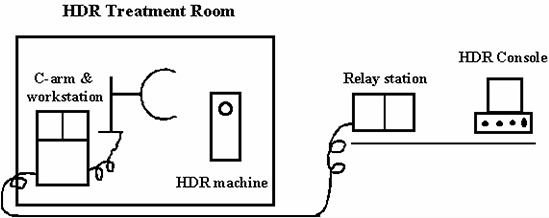
Schematic diagram showing the C‐arm and its dual monitor workstation in the HDR treatment room. The relay station is shown outside the HDR treatment room near the console. One of the monitors of the workstation as well as the relay station is used to display the treatment planning image. The other monitor is used for verification image. A C‐arm remote control pendant is available at the relay station for taking fluoroscopic images of the HDR check cable.

The C‐arm workstation monitors are located inside the HDR treatment room. In order to display images outside the room, two fast scan video monitors (model M15LV‐65MAX, Image Systems Corporation, Minnetonka, Minnesota) are purchased as a relay station for the workstation monitors. The relay monitors are placed at the HDR treatment console, outside the room, and their inputs are connected to the C‐arm workstation video outputs. Fig. 1 is a schematic diagram showing the layout. The C‐arm control pendant is extended to the HDR treatment console, in order to facilitate taking images outside the treatment room.

Before treatment delivery, the treatment planning image is displayed on one of the monitors of the workstation and the relay station, and the intended first and last dwell positions of the source are marked using annotation tools available from the C‐arm workstation. Upon initiation of the HDR treatment sequence, the check cable is sent to the first, i.e., most distal dwell position inside the implant catheter. As a safety feature, the check cable momentarily overshoots the first dwell position by several millimeters. It then retracts to and dwells at the first dwell position for 3 seconds. During the dwell period, a snapshot of the check cable is taken with the C‐arm fluoroscopic machine, using the remote pendant at the treatment console. This verification image is automatically displayed on the second monitor of the relay station. At this point, the operator interrupts the HDR treatment sequence, and the verification image is evaluated. The treatment is started if the tip of the check cable on the verification image is at the intended first dwell position, as indicated on the treatment planning image.

For implants with more than one catheter, a separate fluoroscopic image snapshot is required for each catheter. The check cable's travel time between the most distal dwell positions in successive catheters is about 7 seconds. This is in addition to the 3 seconds dwell time at each of the most distal dwell positions. This amount of time is sufficient to allow for a quick visual inspection of the verification image, and to save it to the workstation using the remote pendant at the console. Once all verification images are taken, the HDR treatment sequence is interrupted, and a detailed evaluation of all images is conducted. Ideally, the C‐arm workstation software could make a composite image of all the individual verification images. Such a composite image would provide the verification of the positions for all the catheters in a short time. Unfortunately, the C‐arm workstation used in this study does not provide such functionality.

## III. RESULTS AND DISCUSSION

**Figure 2:**
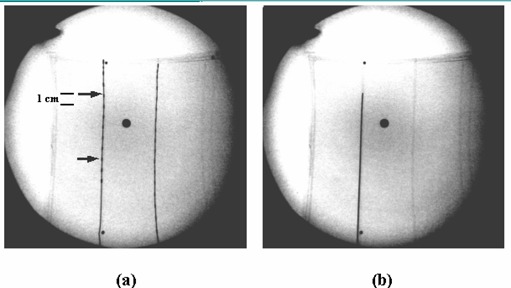
Application of our verification method to a two‐catheter implant in a phantom. (a) Treatment planning image taken in the AP orientation with the first and the last dwell positions of the source marked using arrows. The distance scale is indicated by the dummy seeds, spaced 1 cm apart, and is marked in the figure. (b) Verification image of the check cable at the first dwell position compared to the first dwell position in Fig. 2(a).

Fig. 2 shows the results of the application of our verification method, for an implant with two catheters in a phantom. Fig. 2(a) is the anterior‐posterior (AP) image of the phantom with dummy ribbons loaded in the catheters. This image, together with a lateral (LAT) image, is used for treatment planning, and is displayed on one of the monitors of the C‐arm workstation and relay station (see Fig. 1). The two arrows in the image indicate the first and the last dwell positions for the catheter on the left. The distance scale is indicated by the dummy seeds, spaced 1 cm apart, and is marked in the figure. Fig. 2(b) is the verification image of the check cable, as it reaches the first dwell position in the left catheter. With both the images displayed side‐by‐side on the dual monitors at the treatment console, any discrepancy between the intended first dwell position on the planning image and the tip of the check cable on the verification image can be identified easily. Certainly, verification of the second catheter on the right requires a separate fluoroscopic image snapshot, as the check cable reaches that catheter.

**Figure 3:**
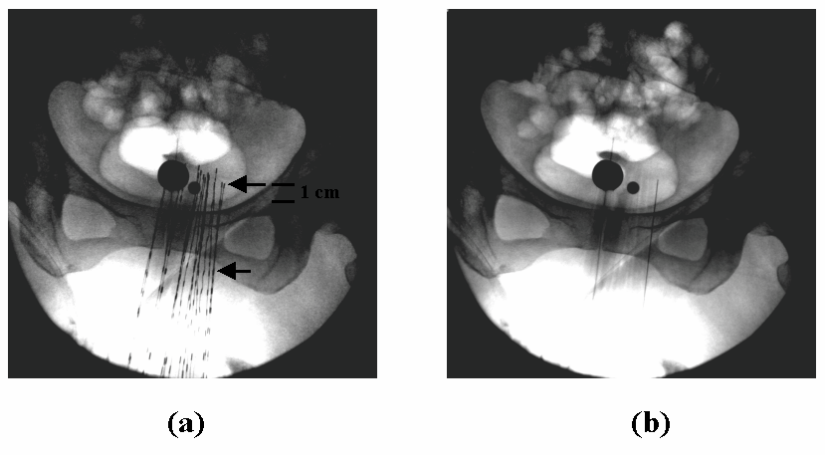
Example of our verification method applied to a 16‐catheter interstitial gynecological implant. (a) Reference image taken in the AP orientation with the first and last dwell positions for the first catheter marked. The distance scale is indicated by the dummy seeds spaced at 1 cm apart and is marked in the figure. (b) Verification image of the check cable at the first dwell position of the first catheter.

For endobronchial and intracavitary implants, the C‐arm fluoroscopic machine provides a convenient and practical solution as a treatment planning imaging device.^(4)^ The application of the dwell position verification method is similar to the example of Fig. 2. When other imaging devices, such as the computed tomography (CT) scanner or simulator, are used for HDR brachytherapy treatment planning, a separate C‐arm fluoroscopic image is taken prior to the treatment delivery. This can be done with or without dummy seed ribbons loaded in the implant catheters. The intended dwell positions obtained from the CT or simulator planning are marked on the C‐arm image, and this image is used as the planning or reference image for comparison with the verification image. Fig. 3 is an example of such a clinical application to an interstitial gynecological implant using the Syed template. Dummy ribbons are loaded in the 16‐catheter implant for the reference image, as shown in Fig. 3(a). The first and the last dwell positions of the first catheter are marked, as indicated by the two arrows. The distance scale is indicated by the dummy seeds, spaced 1 cm apart, and is marked in the figure. Fig. 3(b) is the verification image for the first catheter.

For endobronchial and intracavitary implants, the extra time required to set up the fluoroscopic system and to complete the verification process is about 2 minutes, if performed by trained personnel. For interstitial implants using CT or simulator images, the extra time required is 5–10 minutes depending on the number of catheters in the implant. Such a small amount of time spent on the verification of an important treatment parameter is well justified.

It is worth pointing out that dwell position verification is not meant to replace catheter depth measurements that require millimeter accuracy. Rather, the purpose of the verification is to catch any gross errors in the determination of the dwell positions during the treatment planning process. One can use the dummy ribbons in the reference image and patient anatomic landmarks as a distance‐measuring tool. In both Figs. 2(a) and 3(a), the distance between adjacent dummy seeds is 1 cm. Any position discrepancy of 1 cm or greater can be noticed, immediately, by visual inspection of the images, and smaller ones, with the aid of a ruler and the reference landmarks on the images. In order to facilitate image comparison, one can also install a graticule on the image intensifier. However, such a device tends to obstruct the dummy ribbons in the treatment planning image, especially for large interstitial implants.

The current verification method is limited to the first dwell position for each implant catheter. The last dwell position, such as that marked by the second arrow in Figs. 2(a) and 3(a), cannot be verified, because the check cable does not dwell at this position. One can, certainly, reprogram the HDR treatment console, so that only the last dwell position in the catheter is kept and a separate check cable run sends the cable to this position. However, this approach requires creating a separate treatment control file, and it is not a direct quality assurance of the actual control file used for the patient treatment delivery.

The implementation of the current method for the verification of dwell position is simple and can be used on HDR machines made by other manufacturers. The capital investment is minimum, beyond that of the mobile C‐arm machine, which also serves as an imaging device for treatment planning in HDR brachytherapy.^(4)^

## IV. CONCLUSIONS

We have described a method to verify the dwell position of the radioactive source during HDR brachytherapy treatments. Examples of the application of our method to a test phantom and an interstitial implant are presented. A mobile C‐arm fluoroscopic machine is used to capture an image of the check cable of the HDR machine at the most distal dwell position. This image is displayed side‐by‐side with the treatment planning image, on a dual monitor relay station at the HDR treatment console. Any significant difference between the tip of the check cable on the verification image and the intended dwell position on the planning image can be easily identified, thus avoiding the possibility of treating the wrong volume. A deviation of 1 cm or larger can be noticed, simply by visual inspection of the images, and smaller ones, with the aid of a ruler and the reference landmarks on the two images.
